# Antibiotic prescribing across age groups in the Kaiser Permanente Northern California population in association with different diagnoses, and with influenza incidence, 2010–2018

**DOI:** 10.1017/S0950268822000371

**Published:** 2022-02-24

**Authors:** Edward Goldstein, Bruce H. Fireman, Nicola P. Klein, Marc Lipsitch, G. Thomas Ray

**Affiliations:** 1 Department of Epidemiology, Center for Communicable Disease Dynamics, Harvard T.H. Chan School of Public Health, Boston, MA 02115 USA; 2 Kaiser Permanente Division of Research, Oakland, CA 94612 USA; 3 Kaiser Permanente Vaccine Study Center, Oakland, CA 94612 USA; 4 Department of Immunology and Infectious Diseases, Harvard T.H. Chan School of Public Health, Boston, MA 02115 USA

**Keywords:** Antibiotics, influenza, children, adults, ear infection, respiratory illness

## Abstract

There is limited information on the volume of antibiotic prescribing that is influenza-associated, resulting from influenza infections and their complications (such as streptococcal pharyngitis and otitis media). Here, we estimated age/diagnosis-specific proportions of antibiotic prescriptions (fills) for the Kaiser Permanente Northern California population during 2010–2018 that were influenza-associated. The proportion of influenza-associated antibiotic prescribing among all antibiotic prescribing was higher in children aged 5–17 years compared to children aged under 5 years, ranging from 1.4% [95% CI (0.7–2.1)] in aged <1 year to 2.7% (1.9–3.4) in aged 15–17 years. For adults aged over 20 years, the proportion of influenza-associated antibiotic prescribing among all antibiotic prescribing was lower, ranging from 0.7% (0.5–1) for aged 25–29 years to 1.6% (1.2–1.9) for aged 60–64 years. Most of the influenza-associated antibiotic prescribing in children aged under 10 years was for ear infections, while for age groups over 25 years, 45–84% of influenza-associated antibiotic prescribing was for respiratory diagnoses without a bacterial indication. This suggests a modest benefit of increasing influenza vaccination coverage for reducing antibiotic prescribing, as well as the potential benefit of other measures to reduce unnecessary antibiotic prescribing for respiratory diagnoses with no bacterial indication in persons aged over 25 years, both of which may further contribute to the mitigation of antimicrobial resistance.

## Introduction

A significant amount of antibiotics is prescribed for illnesses caused by respiratory viruses including influenza [1], with many of those prescriptions being inappropriate [2–4]. Yet, there is limited information on the overall volume of antibiotic prescribing triggered by influenza infections in different age groups. Such information could help guide vaccination efforts and efforts at reducing unnecessary antibiotic prescribing for viral infections without bacterial complications with the aim of reducing antibiotic prescribing and the propagation of antimicrobial resistance [5]. For example, results of a randomised trial of influenza vaccination in children aged 6–36 months suggest that vaccination is associated with a substantial reduction in influenza-related medical utilisation, including decreases in antibiotic prescribing for PCR-confirmed influenza episodes [6]; however, it is uncertain how much influenza vaccination would affect the overall volume of antibiotic prescribing in different age groups. A statistical analysis of data on antibiotic prescriptions to Scottish children aged under 5 years between 2009 and 2017 estimated that 2.4% of those prescriptions are influenza-associated [7]. A French study covering a 7-year period found that during the cold seasons, outpatient visits related to influenza-like illness (ILI) contributed between 5% and 10% of all outpatient antibiotic prescriptions in children aged under 5 years. However, the estimates in [8] refer to antibiotic prescriptions during cold seasons and not the entire year (with proportions of antibiotic prescribing that are influenza-associated being quite lower for the entire year compared to cold seasons), and many ILI episodes have aetiology other than influenza. Moreover, the average weekly rate of antibiotic prescribing to children aged under 5 years during the entire year in a study [8] (3268 prescriptions per week) is quite higher than the antibiotic prescribing rates to younger children in the US, which further suggests higher rates of antibiotic prescribing for respiratory infections including influenza to younger French children compared to younger children in the US. A study of 14 987 outpatients with an acute respiratory infection (ARI) during two influenza seasons in the US found that antibiotic prescribing to persons with laboratory-confirmed influenza accounted for 17% of all antibiotic prescribing for non-pneumonia ARI [1]. However, the contribution of influenza to the overall volume of antibiotic prescribing cannot be estimated from that study. Moreover, the study [1] only refers to ARI episodes during the 4.5-month period in the 2013–2014 influenza season (driven by a novel A/H1N1 variant [9]) and the 5-month period in the 2014–2015 influenza season (a major influenza season driven by a novel A/H3N2 variant [10]) – thus, the average relative contribution of influenza to the volume of outpatient ARI prescribing during the entire year is expected to be much lower.

Individuals prescribed antibiotics for respiratory and other diagnoses are rarely tested for influenza, and the volume of influenza-associated antibiotic prescribing cannot be estimated directly from population-level clinical data. Using a previously developed statistical method [11, 12], we here estimate influenza-associated prescribing (prescribing stemming from influenza infections and their complications) by the proportion of all antibiotic prescribing that can be explained statistically by weekly variation in influenza incidence, and we evaluated this proportion for different diagnoses and different age groups of children and adults in Kaiser Permanente Northern California (KPNC) during 2010–2018. Our estimates are relevant for estimating some of the quantities mentioned above, namely (i) the effect of influenza vaccination on antibiotic prescribing, and the mitigation of antibiotic resistance [5]; (ii) the role of influenza aetiology in unnecessary/inappropriate antibiotic prescribing, particularly for respiratory illness [2–4].

## Methods

Proprietary data on antibiotic prescriptions (fills), related diagnoses, and influenza tests were extracted from the KPNC's Virtual Data Warehouse and Electronic Health Records [13].

### Study population and setting

KPNC is an integrated health care system with a membership of approximately 4 million in 2018, including approximately 3 million members between 4 and 64 years of age. Members receive nearly all their KPNC-related medical care at KPNC facilities (unless referred to outside specialists), which include 46 medical clinics and 21 hospitals [14, 15]. KPNC members comprise more than 30% of the population in Northern California and are representative of the population's racial, ethnic, and socioeconomic distribution, although KPNC somewhat underrepresents those at the very lowest incomes [14, 16].

### Antibiotics and diagnoses

We extracted *all* antibiotic fills, and the diagnoses associated with those prescriptions, for KPNC members between September 2010 and August 2018. Antibiotic fills were those prescriptions that were actually picked up by, or delivered to, the patient. The diagnoses associated with the antibiotic prescriptions were classified into three categories: (1) all diagnoses; (2) ear infections; (3) respiratory diagnoses without an indication of a bacterial infection (such as a diagnosis of streptococcal pharyngitis) – see Tables S1 and S2 in the Supplementary Material for more details on diagnoses in categories (2), (3). The reason for considering respiratory diagnoses without an indication of a bacterial infection is that antibiotics are prescribed frequently and often inappropriately for such illness episodes [2–4], and evaluation of the role of influenza aetiology for such antibiotic prescribing should further help to support the case for avoiding unnecessary prescribing for respiratory illness without a bacterial indication. Further details on the motivation behind the examination of diagnosis categories (1)–(3) are provided in the Discussion. Using these data, we calculated weekly rates of antibiotic prescriptions (fills) for the different categories of diagnoses in different age groups. The denominator for the rates of antibiotic prescribing was the KPNC weekly member-time for each of the respective age groups.

### Influenza incidence

Weekly rates of respiratory tests positive for influenza A and for influenza B per 100 000 Kaiser Permanente members in different age groups were used as *incidence proxies* for influenza A and B in different age groups. The California Department of Health data for Public Health labs [17] for the San Francisco Bay Area and Northern California were used to estimate the (weekly) relative share of influenza A/H1N1 and A/H3N2 among influenza A specimens. Those weekly relative shares of influenza A/H3N2 and A/H1N1 among influenza A specimens were multiplied by the incidence proxies for influenza A described above to define incidence proxies for influenza A/H3N2 and A/H1N1 in different age groups.

### Statistical inference

To estimate the volume of influenza-associated antibiotic prescribing for different diagnoses and age groups, we performed separate inference for antibiotic prescriptions for each of the three above categories of diagnoses in each of the 23 selected age groups (Results). Adapting previously developed methodology [11, 12], weekly rates of antibiotic prescribing for different diagnosis categories in different age groups were regressed linearly on the age-specific incidence proxies for the major influenza subtypes (A/H3N2, A/H1N1 and B), periodic weekly rates of antibiotic prescribing (baseline seasonality) that are not associated with influenza circulation modelled by trigonometric (sine and cosine) functions with annual periodicity and temporal trend terms (quadratic polynomials in week). In the regression model, we put a lag of up to one week between influenza incidence proxies and the rates of associated antibiotic prescriptions (eq. 1) under the assumption that there is the time between illness episodes and fills at Kaiser Permanente pharmacies for prescriptions for complications stemming from influenza infections.

During our study period, the circulating influenza A/H3N2 strains were replaced by a genetically different lineage during the 2014–2015 season that rendered previously used vaccines ineffective [10]. We, therefore, split the incidence proxy for influenza A/H3N2 into two separate proxies for the periods before and after the start of the 2014–2015 season. Similarly, starting from the 2013–2014 season, influenza A/H1N1 circulation was driven by novel strains [9]. Accordingly, we split the incidence proxy for influenza A/H1N1 into two proxies for the periods before and after the start of the 2013–2014 season.

With the incidence proxies for the major influenza subtypes defined above and split into different time periods, the model equation is as follows: let *R*(*t*) be the weekly rates of prescribing of antibiotics for a given category of diagnoses (including all prescribing) per 100 000 persons in a given age group. We linearly regress
1
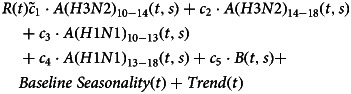


Here, for the covariates related to influenza incidence proxies, *A*(*H*3*N*2)_10−14_(*t*, *s*) is the incidence proxy for influenza A/H3N2 during the 2010–2014 period (so the proxy is set to zero for weeks beginning after 1 September 2014) lagged up to 1 week, so that *A*(*H*3*N*2)_10−14_(*t*,*s*) = *s* ⋅ *A*(*H*3*N*2)_10−14_(*t*) + (1 − *s*) ⋅ *A*(*H*3*N*2)_10−14_(*t* − 1), with the number 0 ≤ *s* ≤ 1 (used in the linear interpolation for the incidence proxies on two consecutive weeks) chosen to minimise the Akaike information criterion (AIC) score of the linear regression model in eq. 1. The covariates *Baseline Seasonality*(*t*) and *Trend*(*t*) are described in the 1st paragraph of this section.

Using this regression framework, we estimate the average annual rates of influenza-associated antibiotic prescribing for the studied categories of diagnosis in different age groups of individuals during 2010–2011 through the 2017–2018 influenza seasons. Influenza-attributable prescribing is estimated by the difference between the predicted value of the dependent variable (fills) with the observed levels of influenza incidence proxies to the predicted value when those proxies are set to zero, with confidence intervals that account for uncertainty in the regression coefficients. Confidence bounds for the model estimates are bootstrapped to account for potential residual auto-correlation as described in [11]. We also estimate the proportion of the overall antibiotic prescribing for those diagnoses in different age groups that are influenza-associated, with proportions of antibiotic prescribing that are influenza-associated being more representative nationally than the prescribing rates themselves.

## Results

### Children

#### Overall antibiotic prescribing

Rates of annual overall antibiotic prescribing per 1000 children during the study period ranged from 375 in children aged 10–14 years to 974 in children aged 1 year (12–23 months), Table 1. The estimated annual rates of influenza-associated antibiotic prescribing per 1000 children ranged from 9.1 (5–13) in aged 10–14 years to 18.1 (8.9–27.4) in aged 1 year. The proportion of influenza-associated antibiotic prescribing among all antibiotic prescribing was higher in children aged 5–17 years compared to children aged under 5 years, ranging from 1.4% [95% CI (0.7–2.1)] in aged <1 year to 2.7% (1.9–3.4) in aged 15–17 years.

**Table 1.** Overall and influenza-associated annual antibiotic prescribing rates per 1000 in different age groups of children, 2010–2018

Age (*y*)/Rate	Overall annual prescribing per 1000	Influenza-associated annual prescribing per 1000	Percent of prescribing that is influenza-associated
<1	812	11.3 (5.7–17)	1.4% (0.7–2.1)
1	974	18.1 (8.9–27.4)	1.9% (0.9–2.8)
2	772	15.9 (8.1–23.7)	2.1% (1–3.1)
3	715	14 (6.5–21.6)	2% (0.9–3)
4	660	11.5 (4.4–18.6)	1.7% (0.7–2.8)
5–9	487	12.9 (5.6–20)	2.7% (1.2–4.1)
10–14	375	9.1 (5–13)	2.4% (1.3–3.5)
15–17	602	16.3 (11.4–20.7)	2.7% (1.9–3.4)

#### Antibiotic prescribing for ear infection and respiratory diagnoses without a bacterial indication

Rates of antibiotic prescribing for ear infections generally declined with age, ranging from 24.2 per 1000 children aged 15–17 years to 430.6 per 1000 children aged 1 year (Table 2). Rates of antibiotic prescribing for respiratory diagnoses without a bacterial indication ranged from 57.8 per 1000 children aged <1 year to 132.9 per 1000 children aged 4 years (Table 2). The proportion of antibiotic prescribing for ear infections that was influenza-associated ranged from 3.1% (1.9–4.2) for children aged under 1 year to 9.2% (6.2–12.3) for children aged 15–17 years (Table 2). Rates of influenza-associated antibiotic prescribing for ear infections in children aged under 10 years were quite high (highest in children aged 1 year), with influenza-associated antibiotic prescribing for ear infections accounting for most of the overall influenza-associated antibiotic prescribing in those children (Table 2 *vs.* Table 1). The proportion of antibiotic prescribing for respiratory diagnoses without a bacterial indication that was influenza-associated ranged from 0.8% (0–2.3) for children aged 4 years to 4% (1.5–6.5) for children aged 15–17 years (Table 2), with rates of influenza-associated antibiotic prescribing for respiratory diagnoses without a bacterial indication being higher in children aged over 10 years *vs.* children aged under 10 years (Table 2).

**Table 2.** Overall and influenza-associated annual antibiotic prescribing rates for ear infections and for respiratory diagnoses without a bacterial indication per 1000 in different age groups of children, 2010–2018

Age (*y*)/Rate	Ear infections, annual prescribing per 1000	Ear infections, flu-associated annual prescribing per 1000	Percent of prescribing that is flu-associated	Respiratory diagnoses without a bacterial indication, annual prescribing per 1000	Respiratory diagnoses without a bacterial indication, annual flu-associated prescribing per 1000	Percent of prescribing that is flu-associated
<1	298.9	9.2 (5.8–12.6)	3.1% (1.9–4.2)	57.8	0.8 (0–1.6)	1.3% (0–2.7)
1 (12–23 months)	430.6	13.9 (8.4–19.5)	3.2% (2–4.5)	113.7	1.1 (0–2.5)	1% (0–2.2)
2	271.3	10.9 (6.8–15.1)	4% (2.5–5.6)	122.5	2(0.4–3.6)	1.7% (0.3–3)
3	226.8	9.9 (6.1–13.7)	4.4% (2.7–6.1)	131.1	1.4(0–3)	1.1% (0–2.3)
4	193	8.6 (5.1–12.2)	4.5% (2.6–6.3)	132.9	1.1 (0–3)	0.8% (0–2.3)
5–9	101.5	9.2 (6.2–12.1)	9% (6.1–12)	118.7	1.4 (0–3.5)	1.2% (0–3)
10–14	38.4	3.1 (1.9–4.2)	8% (5.1–11)	78.6	2.4 (0.1–4.8)	3.1% (0.1–6)
15–17	24.2	2.2 (1.5–3)	9.2% (6.2–12.3)	83.1	3.3 (1.2–5.4)	4% (1.5–6.5)

### Adults aged 18–59 years

Rates of annual overall antibiotic prescribing per 1000 younger/middle-aged adults during the study period ranged from 526 in aged 20–24 years to 658 in aged 55–59 years (Table 3). The proportion of overall antibiotic prescribing that was influenza-associated ranged from 0.8% (0.5–1.2) in those aged 30–34 years to 2.4% (1.3–3.5) in those aged 18–19 years. (Table 3). Rates of influenza-associated antibiotic prescribing were highest in adults aged 18–19 years, followed by those aged 55–59 years. Between 49% and 84% of influenza-associated antibiotic prescribing in age groups of adults over 25 years was for respiratory diagnoses without a bacterial indication. The proportion of antibiotic prescribing for respiratory diagnoses without a bacterial indication that was influenza-associated ranged from 2.7% (1.4–4) in those aged 25–29 years to 6.2% (4.8–7.7) in those aged 45–49 years (Table 3).

**Table 3.** Overall and influenza-associated annual antibiotic prescribing rates per 1000 in different age groups of younger/middle-aged adults, as well as prescribing for respiratory diagnoses without a bacterial indication, 2010–2018

Age (*y*)/Rate	Overall annual prescribing per 1000	Flu-associated annual prescribing per 1000	Percent of prescribing that is flu-associated	Respiratory diagnoses without a bacterial indication, annual prescribing per 1000	Respiratory diagnoses without a bacterial indication, annual flu-associated prescribing per 1000	Percent of prescribing that is flu-associated
18–19	563	13.2 (7–19.6)	2.4% (1.3–3.5)	68.6	2.5 (1.6–3.4)	3.7% (2.4–5)
20–24	526	7.5 (5.3–9.7)	1.4% (1–1.8)	62.5	2 (1.3–2.7)	3.2% (2.1–4.4)
25–29	556	4.1 (2.5–5.5)	0.7% (0.5–1)	73.3	2 (1.1–2.9)	2.7% (1.4–4)
30–34	560	4.5 (2.9–5.9)	0.8% (0.5–1.1)	86.8	2.4 (1.2–3.6)	2.8% (1.4–4.2)
35–39	567	5.6 (4–7.2)	1% (0.7–1.3)	97.3	4.1 (3.1–5.1)	4.2% (3.1–5.3)
40–44	560	8 (5.6–10.2)	1.4% (1–1.8)	97.4	4.6 (3.5–5.7)	4.7% (3.6–5.8)
45–49	571	7.1 (5.3–8.8)	1.2% (0.9–1.5)	96.6	6 (4.6–7.4)	6.2% (4.8–7.7)
50–54	609	8.8 (6.5–10.9)	1.4% (1.1–1.8)	101.6	5.8 (4.5–7.1)	5.7% (4.4–7)
55–59	658	10.1 (7.7–12.3)	1.5% (1.2–1.9)	108.7	6.5 (5.1–7.9)	6% (4.7–7.3)

### Adults aged over 60 years

Rates of annual overall antibiotic prescribing per 1000 older adults during the study period increased with age, from 724 in those aged 60–64 years to 1208 in those aged over 85 years (Table 4). The proportion of overall antibiotic prescribing that was influenza-associated was relatively stable across age groups of older adults, ranging from 1.1% (0.8–1.3) in those aged 75–79 years to 1.6% (1.2–1.9) in those aged 60–64 years (Table 4). Rates of influenza-associated antibiotic prescribing were highest in the oldest adults, reaching 18.5 (13.7–22.9) annual prescriptions per 1000 adults aged over 85 years. Between 45% and 62% of influenza-associated antibiotic prescribing in age groups of adults over 60 years was for respiratory diagnoses without a bacterial indication. The proportion of antibiotic prescribing for respiratory diagnoses without a bacterial indication that was influenza-associated ranged from 4.7% (3.8–5.7) in those aged 65–69 years to 6.6% (5.4–7.8) in those aged over 85 (Table 4).

**Table 4.** Overall and influenza-associated annual antibiotic prescribing rates per 1000 in different age groups of older adults, as well as prescribing for respiratory diagnoses without a bacterial indication, 2010–2018

Age (*y*)/rate	Overall annual prescribing per 1000	Flu-associated annual prescribing per 1000	Percent of prescribing that is flu-associated	Respiratory diagnoses without a bacterial indication, annual prescribing per 1000	Respiratory diagnoses without a bacterial indication, annual flu-associated prescribing per 1000	Percent of prescribing that is flu-associated
60–64	724	11.3 (8.6–13.7)	1.6% (1.2–1.9)	113	6.6 (5.3–8)	5.9% (4.7–7.1)
65–69	831	12.4 (9.3–15.3)	1.5% (1.1–1.8)	119.4	5.6 (4.5–6.8)	4.7% (3.8–5.7)
70–74	939	13.9 (10.1–17.3)	1.5% (1.1–1.8)	128.6	6.3 (5.1–7.5)	4.9% (4–5.9)
75–79	1031	11.1 (8–13.9)	1.1% (0.8–1.3)	132.7	7.3 (5.9–8.8)	5.5% (4.4–6.6)
80–84	1115	12.9 (9.4–16.2)	1.2% (0.8–1.5)	134.5	8.1 (6.6–9.6)	6% (4.9–7.2)
85 +	1208	18.5 (13.7–22.9)	1.5% (1.1–1.9)	131.8	8.7 (7.1–10.3)	6.6% (5.4–7.8)

## Discussion

Influenza vaccination coverage in different age groups in the US has increased gradually with time [18]. Further increases in vaccination coverage have the potential to further mitigate influenza epidemics and influenza-related outcomes, including antibiotic prescribing. However, our understanding of the contribution of influenza to antibiotic prescribing in different age groups is limited. In this study, we evaluated the contribution of influenza to antibiotic prescribing for different indications in different age groups of children and adults in KPNC between 2010 and 2018. This contribution is related to both the effect of influenza vaccination on overall antibiotic prescribing, and the role of influenza aetiology in unnecessary/inappropriate antibiotic prescribing, particularly for respiratory illness [2–4]. We found that for children aged under 18 years, the relative contribution of influenza to the overall volume of antibiotic prescribing was higher in children aged 5–17 years compared to children aged under 5 years, ranging from 1.4% [95% CI (0.7–2.1)] in aged <1 year to 2.7% (1.9–3.4) in aged 15–17 years. For adults aged over 20 years, the proportion of influenza-associated antibiotic prescribing among all antibiotic prescribing was lower than for children, ranging from 0.7% (0.5–1) for aged 25–29 years to 1.6% (1.2–1.9) for aged 60–64 years. Overall, our findings support the notion that the benefit of influenza vaccination for reducing the volume of antibiotic prescribing is expected to be modest overall, with that benefit being greatest in school-age children. Most of the influenza-associated antibiotic prescribing in children aged under 10 years was for ear infections, while in age groups of adults over 25 years, 45–84% of influenza-associated antibiotic prescribing was for respiratory diagnoses without a bacterial indication. The estimates of the contribution of influenza to antibiotic prescribing for respiratory diagnoses without a bacterial indication in persons aged over 25 years are important not only in terms of reducing influenza-associated antibiotic prescribing through vaccination but also in terms of reducing antibiotic prescribing for respiratory illness not involving evidence of a bacterial infection, with antibiotics being prescribed frequently and often inappropriately for such illness diagnoses [2–4]. We also note in that regard that the estimated relative contribution of influenza to antibiotic prescribing for respiratory illness not involving evidence of a bacterial infection generally increased with age (Tables 2–4), which suggests that the likelihood of influenza-associated antibiotic prescribing for respiratory illness not involving evidence of a bacterial infection is increasing with age, and is highest for older adults. Finally, our estimates of the rates of influenza-associated antibiotic prescribing for ear infections in Table 2, which are quite high for children aged under 10 years provide further support for the benefit of wider influenza vaccination coverage for children for reducing antibiotic prescribing for ear infections in children and reducing the volume of illness related to ear infections, particularly acute otitis media (AOM) in younger children.

Our estimates of the relative contribution of influenza to the overall volume of antibiotic prescribing in children aged under 5 years (around 1.8%) are somewhat lower than the corresponding estimate (2.4%) in Scotland [7]. Those differences might be potentially related to differences in the practices of antibiotic prescribing in the KPNC healthcare compared to the UK, with the latter practices in the UK changing with time [19]. We note that we estimated that around 2.6% of antibiotic prescribing to school-age children (aged 5–17 years) in the Kaiser Permanente population was influenza-associated. Influenza infection was detected in 4.4% of AOM episodes (25/566) in children in [20], and 5.3% of AOM episodes in children (24/456) in [21], both studies extending throughout the year (not only influenza season). These findings are in good agreement with our estimates of the contribution of influenza to antibiotic prescribing for ear infections in children (Table 2).

Compared to estimates in children, e.g. studies [7, 20, 21], less is known about the relative contribution of influenza to antibiotic prescribing in adults, though this contribution is expected to be lower than the relative contribution of influenza to antibiotic prescribing in children because (i) influenza generally circulates more actively in children compared to adults (e.g. [22]); (ii) the relative share of antibiotic prescribing for respiratory causes and ear infections among all antibiotic prescribing is higher in children than in adults – compare Tables 1 and 2 *vs.* Tables 3 and 4 (with very limited antibiotic prescribing for ear infections in adults). This is consistent with our finding that the proportion of overall antibiotic prescribing that is influenza-associated is generally lower in adults than in children (Table 1 *vs.* Tables 3 and 4).

Our inference method has limitations, primarily in not being able to account for year-to-year variability in antibiotic prescribing associated with circulating viruses other than influenza, including the respiratory syncytial virus. Further work is needed to assess the contribution of different respiratory viruses to antibiotic prescribing, including the contribution of influenza to antibiotic prescribing in adults. Prescribing patterns at Kaiser Permanente may not be broadly generalisable because KPNC frequently tests for influenza; for example, physicians elsewhere may prescribe antibiotics for visits stemming from influenza infections (particularly undetected influenza infections) more frequently. Around 22% of all antibiotic prescriptions in the KPNC dataset are missing a diagnosis. This has no effect on the estimates of the contribution of influenza to overall antibiotic prescribing. It should also not bias the estimates of the proportion of antibiotic prescribing for respiratory illness without a bacterial indication and for ear infections that is influenza-associated (unless the likelihood of missing those diagnoses is different for influenza-associated cases *vs.* cases that were not influenza-associated). Some antibiotic prescriptions issued to KPNC members may not be recorded in the KPNC database. However, KPNC has a closed pharmacy system that ensures virtually complete capture of prescription drug dispensing. Patients with KPNC prescription drug benefits must purchase their medications at one of ~120 walk-in pharmacies or, since 1999, via mail order [23]. While it is possible to transfer a prescription to an out-of-plan pharmacy, a recent study in a similar patient population (Kaiser Permanente Colorado) found that the prevalence of out-of-plan pharmacy prescription transfers was still fairly low in 2011 (about 5%) even after the widespread introduction of bargain generic prescription programs beginning in 2006 [23, 24]. Moreover, even if a fraction of antibiotic prescriptions issued to individuals who are KPNC members (including prescriptions by doctors who are not part of the KPNC healthcare) is not included in the dataset that we've used, this shouldn't bias the estimates of the proportion of antibiotic prescriptions that are influenza-associated unless influenza-associated antibiotic prescriptions have a different likelihood of being un-recorded in the KPNC database compared to antibiotic prescriptions that are not influenza-associated. Finally, for respiratory diagnoses without a bacterial indication, some of those illness episodes may actually involve (undiagnosed) bacterial infections, and the contribution of influenza aetiology to antibiotic prescribing is expected to be higher for respiratory diagnoses for which undetected bacterial infection is less likely. Thus, the estimates for respiratory diagnoses without a bacterial indication in our paper are meant to be conservative, namely that the relative contribution of influenza to antibiotic prescribing for respiratory illness with an unlikely bacterial infection may be (somewhat) higher than the corresponding estimates for respiratory diagnoses without a bacterial indication in our paper.

Our results provide estimates of the relative contribution of influenza to antibiotic prescribing for different diagnoses in different age groups which are compatible with related findings in the literature. Our results suggest an overall modest benefit of increasing influenza vaccination coverage for reducing antibiotic prescribing, with that benefit being greatest for school-age children, as well as the benefit of reducing unnecessary antibiotic prescribing for respiratory diagnoses with no bacterial indication in persons aged over 25 years, both of which may further contribute to the mitigation of antimicrobial resistance.

## Data Availability

Data on the circulation of influenza subtypes in the San Francisco Bay Area/Northern California between 2010 and 2018 are publicly available from the California Department of Public Health, and can be accessed at https://data.chhs.ca.gov/dataset/influenza-surveillance.

## Supplementary material

For supplementary material accompanying this paper visit http://doi.org/10.1017/S0950268822000371. click here to view supplementary material

## References

[1] Havers FP (2018) Outpatient antibiotic prescribing for acute respiratory infections during influenza seasons. Journal of the American Medical Association Network Open 1, e180243. 10.1001/jamanetworkopen.2018.0243 PMC6324415 30646067

[2] Fleming-Dutra KE (2016) Prevalence of inappropriate antibiotic prescriptions among US ambulatory care visits, 2010–2011. Journal of the American Medical Association 315, 1864–1873. 27139059 10.1001/jama.2016.4151

[3] Hagedoorn NN (2020) Variation in antibiotic prescription rates in febrile children presenting to emergency departments across Europe (MOFICHE): a multicentre observational study. PLoS Medicine 17, e1003208. 32813708 10.1371/journal.pmed.1003208 PMC7444592

[4] Silverman M (2017) Antibiotic prescribing for nonbacterial acute upper respiratory infections in elderly persons. Annals of Internal Medicine 166, 765–774. 28492914 10.7326/M16-1131

[5] Klugman KP and Black S (2018) Impact of existing vaccines in reducing antibiotic resistance: primary and secondary effects. Proceedings of the National Academy of Sciences of the United States of America 115, 12896–12901. 30559195 10.1073/pnas.1721095115 PMC6304973

[6] Dbaibo G (2020) Quadrivalent influenza vaccine prevents illness and reduces healthcare utilization across diverse geographic regions during five influenza seasons: a randomized clinical trial. The Pediatric Infectious Disease Journal 39, e1–e10. 31725115 10.1097/INF.0000000000002504 PMC7004464

[7] Fitzpatrick T (2021) Community-based antibiotic prescribing attributable to respiratory syncytial virus and other common respiratory viruses in young children: a population-based time-series study of Scottish children. Clinical Infectious Diseases 72, 2144–2153. 32270199 10.1093/cid/ciaa403

[8] Cheysson F (2021) Outpatient antibiotic use attributable to viral acute lower respiratory tract infections during the cold season in France, 2010–2017. International Journal of Antimicrobial Agents 57, 106339. 33852933 10.1016/j.ijantimicag.2021.106339

[9] Linderman SL (2014) Potential antigenic explanation for atypical H1N1 infections among middle-aged adults during the 2013–2014 influenza season. Proceedings of the National Academy of Sciences of the United States of America 111, 15798–15803. 25331901 10.1073/pnas.1409171111 PMC4226110

[10] Skowronski DM (2016) A perfect storm: impact of genomic variation and serial vaccination on low influenza vaccine effectiveness during the 2014–2015 season. Clinical Infectious Diseases 63, 21–32. 27025838 10.1093/cid/ciw176 PMC4901864

[11] Goldstein E (2012) Improving the estimation of influenza-related mortality over a seasonal baseline. Epidemiology (Cambridge, Mass.) 23, 829–838. 10.1097/EDE.0b013e31826c2dda PMC3516362 22992574

[12] Quandelacy TM (2014) Age- and sex-related risk factors for influenza-associated mortality in the United States between 1997–2007. American Journal of Epidemiology 179, 156–167. 24190951 10.1093/aje/kwt235 PMC3873104

[13] Ross TR (2014) The HMO research network virtual data warehouse: a public data model to support collaboration. eGEMs: The Journal for Electronic Health Data and Methods 2, 1049. 10.13063/2327-9214.1049 PMC4371424 25848584

[14] Gordon N and Lin T (2016) The Kaiser Permanente Northern California adult member health survey. The Permanente Journal 20, 34–42. 10.7812/TPP/15-225 PMC5101088 27548806

[15] Selby JV (2005) Kaiser Permanente medical care program. In Strom BL (ed.), Pharmacoepidemiology, 4th Edn. Chichester, West Sussex, UK: John Wiley and Sons, pp. 241–259.

[16] Gordon NP (2020) Similarity of adult Kaiser Permanente members to the insured adult population in Kaiser Permanente's Northern California market: comparisons based on the 2017/2018 cycle of the California Health Interview Survey. Available at https://divisionofresearch.kaiserpermanente.org/projects/memberhealthsurvey/SiteCollectionDocuments/compare_kp_ncal_chis2017-18.pdf (Accessed 24 May 2021).

[17] California Department of Public Health (2022) Available at: http://cdphready.org/the-california-department-of-public-health-cdph-influenza-surveillance-program/ (Accessed February 4, 2022).

[18] US Centers for Disease Control and Prevention (2022) Available at: https://www.cdc.gov/flu/fluvaxview/coverage-1920estimates.htm (Accessed March 4, 2022).

[19] Chae C (2020) Effect of pediatric influenza vaccination on antibiotic resistance, England and Wales. Emerging Infectious Diseases 26, 138–142. 31574242 10.3201/eid2601.191110 PMC6924886

[20] Patel JA (2007) Role of respiratory syncytial virus in acute otitis media: implications for vaccine development. Vaccine 25, 1683–1689. 17156899 10.1016/j.vaccine.2006.10.045 PMC1828634

[21] Heikkinen T (1999) Prevalence of various respiratory viruses in the middle ear during acute otitis media. The New England Journal of Medicine 340, 260–264. 9920949 10.1056/NEJM199901283400402

[22] Monto AS (1985) Tecumseh study of illness. XIII. Influenza infection and disease, 1976–1981. American Journal of Epidemiology 121, 811–822. 4014174 10.1093/oxfordjournals.aje.a114052

[23] Parker MM (2015) An algorithm to identify medication nonpersistence using electronic pharmacy databases. Journal of the American Medical Informatics Association 22, 957–961. 26078413 10.1093/jamia/ocv054 PMC5009927

[24] Delate T (2012) Out-of-plan pharmacy use by members of a managed care organization. The Permanente Journal 16, 15–21. 22745611 10.7812/tpp/11-148 PMC3383156

