# Supplementary material for: Antibiotic prescribing across age groups in the Kaiser Permanente Northern California population in association with different diagnoses, and with influenza incidence, 2010–2018
Source: Epidemiol Infect. 2022 Feb 24;150:e85. doi: 10.1017/S0950268822000371 (PMC9074113; doi:10.1017/S0950268822000371)
Supplement: Supplementary file 1 [file S0950268822000371sup001.docx]

*Epidemiology and Infection*

**Antibiotic prescribing across age groups in the Kaiser Permanente Northern California population in association with different diagnoses, and with influenza incidence, 2010-2018**

Edward Goldstein^1^, Bruce H. Fireman^2^, Nicola P. Klein^2,3^, Marc Lipsitch^1,4^, G. Thomas Ray^2^

1. Center for Communicable Disease Dynamics, Department of Epidemiology, Harvard T.H. Chan School of Public Health, Boston, MA 02115 United States
2. Kaiser Permanente Division of Research, Oakland, CA 94612 United States
3. Kaiser Permanente Vaccine Study Center, Oakland, CA 94612 United States
4. Department of Immunology and Infectious Diseases, Harvard T.H. Chan School of Public Health, Boston, MA 02115 United States

**Supplementary Material**

***Section S1: Weekly rates of all antibiotic prescriptions per 100,000 individuals and model fits in select age groups***

Figure S1 presents weekly rates of all antibiotic prescriptions per 100,000 individuals in select age groups of children and adults + model fits during our study period.

***Weekly rates of prescribing of all antibiotics per 100,000 individuals in select age groups (black) and model fits (red)***

**
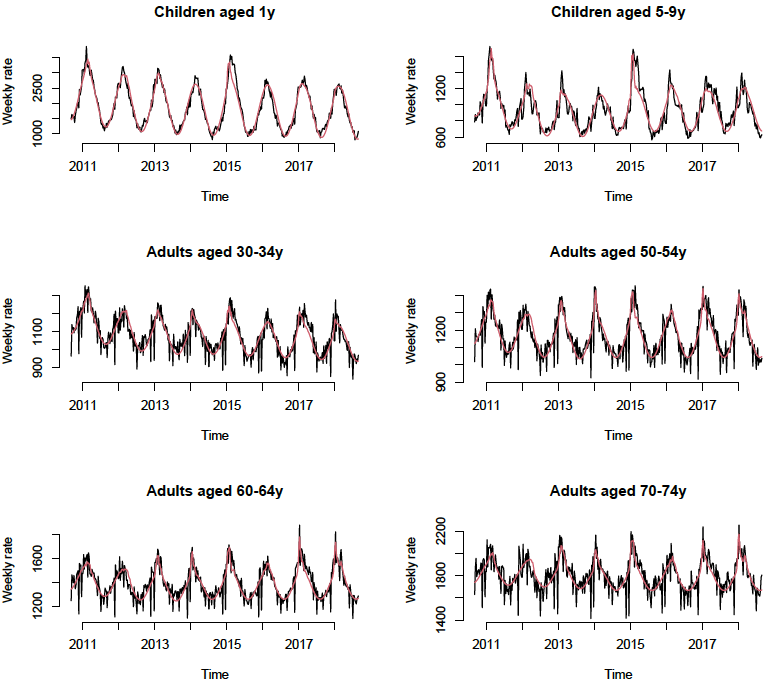
**

**Figure S1**: Weekly rates of all antibiotic prescriptions per 100,000 individuals in select age groups (black) + model fits (red), Sep. 2010-Aug. 2018

Figure S2 presents weekly rates of all antibiotic prescriptions per 100,000 individuals in additional select age groups of children and adults + model fits during our study period.

***Weekly rates of prescribing of all antibiotics per 100,000 individuals in select age groups (black) and model fits (red)***

**Figure S2**: Weekly rates of all antibiotic prescriptions per 100,000 individuals in select age groups (black) + model fits (red), Sep. 2010-Aug. 2018

***Section S2: Diagnoses related to ear infections***

Table S1 lists diagnoses related to ear infections used in the main body of the text.

| ACUTE NONSUPPURATIVE OTITIS MEDIA, UNSPECIFIED | ACUTE SUPPURATIVE OTITIS MEDIA WITH SPONTANEOUS RUPTURE OF EAR DRUM, RECURRENT, BILATERAL |
| --- | --- |
| ACUTE SEROUS OTITIS MEDIA | ACUTE SUPPURATIVE OTITIS MEDIA WITH SPONTANEOUS RUPTURE OF EAR DRUM, RECURRENT, UNSPECIFIED EAR |
| AC MUCOID OTITIS MEDIA | ACUTE SUPPURATIVE OTITIS MEDIA WITH SPONTANEOUS RUPTURE OF EAR DRUM, UNSPECIFIED EAR |
| AC SANGUIN OTITIS MEDIA | CHRONIC TUBOTYMPANIC SUPPURATIVE OTITIS MEDIA, UNSPECIFIED |
| ACUTE ALLERGIC SOM | CHRONIC TUBOTYMPANIC SUPPURATIVE OTITIS MEDIA, RIGHT EAR |
| CHRONIC SOM SIMPLE/NOS | CHRONIC ATTICOANTRAL SUPPURATIVE OTITIS MEDIA, LEFT EAR |
| CHRONIC SOM NEC | OTHER CHRONIC SUPPURATIVE OTITIS MEDIA, RIGHT EAR |
| CHR MUCOID OMED SMPL/NOS | OTHER CHRONIC SUPPURATIVE OTITIS MEDIA, LEFT EAR |
| OTHER AND UNSPECIFIED CHRONIC NONSUPPURATIVE OTITIS MEDIA | OTHER CHRONIC SUPPURATIVE OTITIS MEDIA, BILATERAL |
| NONSUPPURATIVE OTITIS MEDIA, NOT SPECIFIED AS ACUTE OR CHRONIC | OTHER CHRONIC SUPPURATIVE OTITIS MEDIA, UNSPECIFIED EAR |
| EUSTACHIAN SALPING NOS | SUPPURATIVE OTITIS MEDIA, UNSPECIFIED, UNSPECIFIED EAR |
| AC EUSTACHIAN SALPING | SUPPURATIVE OTITIS MEDIA, UNSPECIFIED, RIGHT EAR |
| CHR EUSTACHIAN SALPING | SUPPURATIVE OTITIS MEDIA, UNSPECIFIED, LEFT EAR |
| ACUTE SUPPURATIVE OTITIS MEDIA WITHOUT SPONTANEOUS RUPTURE OF EARDRUM | SUPPURATIVE OTITIS MEDIA, UNSPECIFIED, BILATERAL |
| AC SOM W EARDRUM RUPT | OTITIS MEDIA, UNSPECIFIED, UNSPECIFIED EAR |
| ACUTE SOM IN DCE | OTITIS MEDIA, UNSPECIFIED, RIGHT EAR |
| CHRONIC TUBOTYMPANIC SOM | OTITIS MEDIA, UNSPECIFIED, LEFT EAR |
| CHRONIC ATTICOANTRAL SOM | OTITIS MEDIA, UNSPECIFIED, BILATERAL |
| UNSPECIFIED CHRONIC SUPPURATIVE OTITIS MEDIA | UNSPECIFIED EUSTACHIAN SALPINGITIS, RIGHT EAR |
| UNSPECIFIED SUPPURATIVE OTITIS MEDIA | UNSPECIFIED EUSTACHIAN SALPINGITIS, LEFT EAR |
| UNSPECIFIED OTITIS MEDIA | UNSPECIFIED EUSTACHIAN SALPINGITIS, UNSPECIFIED EAR |
| ACUTE MYRINGITIS, UNSPECIFIED | ACUTE EUSTACHIAN SALPINGITIS, RIGHT EAR |
| BULLOUS MYRINGITIS | ACUTE EUSTACHIAN SALPINGITIS, BILATERAL |
| ACUTE SEROUS OTITIS MEDIA, UNSPECIFIED EAR | CHRONIC EUSTACHIAN SALPINGITIS, LEFT EAR |
| ACUTE SEROUS OTITIS MEDIA, RIGHT EAR | CHRONIC EUSTACHIAN SALPINGITIS, BILATERAL |
| ACUTE SEROUS OTITIS MEDIA, LEFT EAR | ACUTE MYRINGITIS, RIGHT EAR |
| ACUTE SEROUS OTITIS MEDIA, BILATERAL | ACUTE MYRINGITIS, LEFT EAR |
| ACUTE SEROUS OTITIS MEDIA, RECURRENT, RIGHT EAR | ACUTE MYRINGITIS, BILATERAL |
| ACUTE SEROUS OTITIS MEDIA, RECURRENT, LEFT EAR | ACUTE MYRINGITIS, UNSPECIFIED EAR |
| ACUTE SEROUS OTITIS MEDIA, RECURRENT, BILATERAL | BULLOUS MYRINGITIS, RIGHT EAR |
| ACUTE SEROUS OTITIS MEDIA, RECURRENT, UNSPECIFIED EAR | BULLOUS MYRINGITIS, LEFT EAR |
| ACUTE AND SUBACUTE ALLERGIC OTITIS MEDIA (MUCOID) (SANGUINOUS) (SEROUS), RIGHT EAR | BULLOUS MYRINGITIS, BILATERAL |
| ACUTE AND SUBACUTE ALLERGIC OTITIS MEDIA (MUCOID) (SANGUINOUS) (SEROUS), LEFT EAR | BULLOUS MYRINGITIS, UNSPECIFIED EAR |
| ACUTE AND SUBACUTE ALLERGIC OTITIS MEDIA (MUCOID) (SANGUINOUS) (SEROUS), BILATERAL | ACUTE NOM |
| ACUTE AND SUBACUTE ALLERGIC OTITIS MEDIA (MUCOID) (SANGUINOUS) (SEROUS), RECURRENT, LEFT EAR | AC ALLERGIC MUCOID OMED |
| ACUTE AND SUBACUTE ALLERGIC OTITIS MEDIA (MUCOID) (SANGUINOUS) (SEROUS), RECURRENT, BILATERAL | AC ALL SANGUINOUS OMED |
| ACUTE AND SUBACUTE ALLERGIC OTITIS MEDIA (MUCOID) (SANGUINOUS) (SEROUS), UNSPECIFIED EAR | CHRONIC SOM |
| OTHER ACUTE NONSUPPURATIVE OTITIS MEDIA, RIGHT EAR | CHR MUCOID OTITIS MEDIA |
| OTHER ACUTE NONSUPPURATIVE OTITIS MEDIA, LEFT EAR | CHRONIC MUCOID OMED NEC |
| OTHER ACUTE NONSUPPURATIVE OTITIS MEDIA, BILATERAL | EUSTACHIAN SALPINGITIS |
| OTHER ACUTE NONSUPPURATIVE OTITIS MEDIA, UNSPECIFIED EAR | ACUTE MYRINGITIS |
| CHRONIC SEROUS OTITIS MEDIA, UNSPECIFIED EAR | ACUTE MYRINGITIS NEC |
| CHRONIC SEROUS OTITIS MEDIA, RIGHT EAR | ACUTE AND SUBACUTE ALLERGIC OTITIS MEDIA (MUCOID) (SANGUINOUS) (SEROUS), RECURRENT, RIGHT EAR |
| CHRONIC SEROUS OTITIS MEDIA, LEFT EAR | ACUTE AND SUBACUTE ALLERGIC OTITIS MEDIA (MUCOID) (SANGUINOUS) (SEROUS), RECURRENT, UNSPECIFIED EAR |
| CHRONIC SEROUS OTITIS MEDIA, BILATERAL | OTHER ACUTE NONSUPPURATIVE OTITIS MEDIA, RECURRENT, RIGHT EAR |
| CHRONIC MUCOID OTITIS MEDIA, UNSPECIFIED EAR | OTHER ACUTE NONSUPPURATIVE OTITIS MEDIA, RECURRENT, LEFT EAR |
| CHRONIC MUCOID OTITIS MEDIA, RIGHT EAR | OTHER ACUTE NONSUPPURATIVE OTITIS MEDIA, RECURRENT, BILATERAL |
| CHRONIC MUCOID OTITIS MEDIA, LEFT EAR | OTHER ACUTE NONSUPPURATIVE OTITIS MEDIA RECURRENT, UNSPECIFIED EAR |
| CHRONIC MUCOID OTITIS MEDIA, BILATERAL | CHRONIC ALLERGIC OTITIS MEDIA, RIGHT EAR |
| OTHER CHRONIC NONSUPPURATIVE OTITIS MEDIA, RIGHT EAR | CHRONIC ALLERGIC OTITIS MEDIA, LEFT EAR |
| OTHER CHRONIC NONSUPPURATIVE OTITIS MEDIA, LEFT EAR | CHRONIC ALLERGIC OTITIS MEDIA, BILATERAL |
| OTHER CHRONIC NONSUPPURATIVE OTITIS MEDIA, BILATERAL | CHRONIC ALLERGIC OTITIS MEDIA, UNSPECIFIED EAR |
| OTHER CHRONIC NONSUPPURATIVE OTITIS MEDIA, UNSPECIFIED EAR | CHRONIC TUBOTYMPANIC SUPPURATIVE OTITIS MEDIA, LEFT EAR |
| UNSPECIFIED NONSUPPURATIVE OTITIS MEDIA, UNSPECIFIED EAR | CHRONIC TUBOTYMPANIC SUPPURATIVE OTITIS MEDIA, BILATERAL |
| UNSPECIFIED NONSUPPURATIVE OTITIS MEDIA, RIGHT EAR | CHRONIC ATTICOANTRAL SUPPURATIVE OTITIS MEDIA, UNSPECIFIED EAR |
| UNSPECIFIED NONSUPPURATIVE OTITIS MEDIA, LEFT EAR | CHRONIC ATTICOANTRAL SUPPURATIVE OTITIS MEDIA, RIGHT EAR |
| UNSPECIFIED NONSUPPURATIVE OTITIS MEDIA, BILATERAL | CHRONIC ATTICOANTRAL SUPPURATIVE OTITIS MEDIA, BILATERAL |
| ACUTE SUPPURATIVE OTITIS MEDIA WITHOUT SPONTANEOUS RUPTURE OF EAR DRUM, RIGHT EAR | OTITIS MEDIA IN DISEASES CLASSIFIED ELSEWHERE, RIGHT EAR |
| ACUTE SUPPURATIVE OTITIS MEDIA WITHOUT SPONTANEOUS RUPTURE OF EAR DRUM, LEFT EAR | OTITIS MEDIA IN DISEASES CLASSIFIED ELSEWHERE, LEFT EAR |
| ACUTE SUPPURATIVE OTITIS MEDIA WITHOUT SPONTANEOUS RUPTURE OF EAR DRUM, BILATERAL | OTITIS MEDIA IN DISEASES CLASSIFIED ELSEWHERE, BILATERAL |
| ACUTE SUPPURATIVE OTITIS MEDIA WITHOUT SPONTANEOUS RUPTURE OF EAR DRUM, RECURRENT, RIGHT EAR | OTITIS MEDIA IN DISEASES CLASSIFIED ELSEWHERE, UNSPECIFIED EAR |
| ACUTE SUPPURATIVE OTITIS MEDIA WITHOUT SPONTANEOUS RUPTURE OF EAR DRUM, RECURRENT, LEFT EAR | UNSPECIFIED EUSTACHIAN SALPINGITIS, BILATERAL |
| ACUTE SUPPURATIVE OTITIS MEDIA WITHOUT SPONTANEOUS RUPTURE OF EAR DRUM, RECURRENT, BILATERAL | ACUTE EUSTACHIAN SALPINGITIS, LEFT EAR |
| ACUTE SUPPURATIVE OTITIS MEDIA WITHOUT SPONTANEOUS RUPTURE OF EAR DRUM, RECURRENT, UNSPECIFIED EAR | ACUTE EUSTACHIAN SALPINGITIS, UNSPECIFIED EAR |
| ACUTE SUPPURATIVE OTITIS MEDIA WITHOUT SPONTANEOUS RUPTURE OF EAR DRUM, UNSPECIFIED EAR | CHRONIC EUSTACHIAN SALPINGITIS, RIGHT EAR |
| ACUTE SUPPURATIVE OTITIS MEDIA WITH SPONTANEOUS RUPTURE OF EAR DRUM, RIGHT EAR | CHRONIC EUSTACHIAN SALPINGITIS, UNSPECIFIED EAR |
| ACUTE SUPPURATIVE OTITIS MEDIA WITH SPONTANEOUS RUPTURE OF EAR DRUM, LEFT EAR | OTHER ACUTE MYRINGITIS, RIGHT EAR |
| ACUTE SUPPURATIVE OTITIS MEDIA WITH SPONTANEOUS RUPTURE OF EAR DRUM, BILATERAL | OTHER ACUTE MYRINGITIS, LEFT EAR |
| ACUTE SUPPURATIVE OTITIS MEDIA WITH SPONTANEOUS RUPTURE OF EAR DRUM, RECURRENT, RIGHT EAR | OTHER ACUTE MYRINGITIS, BILATERAL |
| ACUTE SUPPURATIVE OTITIS MEDIA WITH SPONTANEOUS RUPTURE OF EAR DRUM, RECURRENT, LEFT EAR | OTHER ACUTE MYRINGITIS, UNSPECIFIED EAR |

**Table S1**: Diagnoses related to ear infections

***Section S3: Respiratory diagnoses without a bacterial indication***

Table S2 lists respiratory diagnoses without an indication of a bacterial infection used in the main body of the text.

| ACUTE NASOPHARYNGITIS [COMMON COLD] | ABSCESS, FURUNCLE AND CARBUNCLE OF NOSE |
| --- | --- |
| AC MAXILLARY SINUSITIS | CYST AND MUCOCELE OF NOSE AND NASAL SINUS |
| AC FRONTAL SINUSITIS | DEVIATED NASAL SEPTUM |
| AC ETHMOIDAL SINUSITIS | HYPERTROPHY OF NASAL TURBINATES |
| ACUTE SPHENOIDAL SINUSITIS | NASAL MUCOSITIS (ULCERATIVE) |
| OTHER ACUTE SINUSITIS | OTHER SPECIFIED DISORDERS OF NOSE AND NASAL SINUSES |
| ACUTE SINUSITIS, UNSPECIFIED | UNSPECIFIED DISORDER OF NOSE AND NASAL SINUSES |
| ACUTE PHARYNGITIS | CHRONIC TONSILLITIS |
| ACUTE TONSILLITIS | CHRONIC ADENOIDITIS |
| ACUTE LARYNGITIS WITHOUT MENTION OF OBSTRUCTION | CHRONIC TONSILLITIS AND ADENOIDITIS |
| AC LARYNGITIS W OBSTR | HYPERTROPHY OF TONSILS |
| AC TRACHEITIS W/O OBSTR | HYPERTROPHY OF ADENOIDS |
| AC TRACHEITIS W OBSTR | HYPERTROPHY OF TONSILS WITH HYPERTROPHY OF ADENOIDS |
| AC LARYNGOTRACH S OBSTR | OTHER CHRONIC DISEASES OF TONSILS AND ADENOIDS |
| AC EPIGLOTTITIS S OBSTR | CHRONIC DISEASE OF TONSILS AND ADENOIDS, UNSPECIFIED |
| AC EPIGLOTTITIS W OBSTR | PERITONSILLAR ABSCESS |
| SUPRAGLOTTITIS W/O OBSTR | CHRONIC LARYNGITIS |
| SUPRAGLOTTITIS W OBSTR | CHRONIC LARYNGOTRACHEITIS |
| ACUTE LARYNGOPHARYNGITIS | PARALYSIS OF VOCAL CORDS AND LARYNX, UNSPECIFIED |
| ACUTE UPPER RESPIRATORY INFECTIONS OF UNSPECIFIED SITE | PARALYSIS OF VOCAL CORDS AND LARYNX, UNILATERAL |
| ACUTE BRONCHITIS | PARALYSIS OF VOCAL CORDS AND LARYNX, BILATERAL |
| AC BRONCHIOLITIS D/T RSV | POLYP OF VOCAL CORD AND LARYNX |
| ACUTE BRONCHIOLITIS DUE TO OTHER INFECTIOUS ORGANISMS | NODULES OF VOCAL CORDS |
| DEVIATED NASAL SEPTUM | OTHER DISEASES OF VOCAL CORDS |
| NASAL CAVITY POLYP | EDEMA OF LARYNX |
| NASAL SINUS POLYP NEC | LARYNGEAL SPASM |
| NASAL POLYP NOS | STENOSIS OF LARYNX |
| CHRONIC RHINITIS | OTHER DISEASES OF LARYNX |
| CHRONIC PHARYNGITIS | RETROPHARYNGEAL AND PARAPHARYNGEAL ABSCESS |
| CHRONIC NASOPHARYNGITIS | OTHER ABSCESS OF PHARYNX |
| CHRONIC MAXILLARY SINUSITIS | OTHER DISEASES OF PHARYNX |
| CHR FRONTAL SINUSITIS | UPPER RESPIRATORY TRACT HYPERSENSITIVITY REACTION, SITE UNSPECIFIED |
| CHR ETHMOIDAL SINUSITIS | OTHER SPECIFIED DISEASES OF UPPER RESPIRATORY TRACT |
| CHR SPHENOIDAL SINUSITIS | DISEASE OF UPPER RESPIRATORY TRACT, UNSPECIFIED |
| CHRONIC SINUSITIS NEC | BRONCHITIS, NOT SPECIFIED AS ACUTE OR CHRONIC |
| UNSPECIFIED SINUSITIS (CHRONIC) | SIMPLE CHRONIC BRONCHITIS |
| CHRONIC TONSILLITIS | MUCOPURULENT CHRONIC BRONCHITIS |
| CHRONIC ADENOIDITIS | UNSPECIFIED CHRONIC BRONCHITIS |
| CHR TONSILLITIS&ADENOID | CENTRILOBULAR EMPHYSEMA |
| HYPERTROPHY OF TONSIL WITH ADENOIDS | EMPHYSEMA, UNSPECIFIED |
| HYPERTROPHY TONSILS | CHRONIC OBSTRUCTIVE PULMONARY DISEASE WITH ACUTE LOWER RESPIRATORY INFECTION |
| HYPERTROPHY OF ADENOIDS ALONE | CHRONIC OBSTRUCTIVE PULMONARY DISEASE WITH (ACUTE) EXACERBATION |
| OTHER CHRONIC DISEASE OF TONSILS AND ADENOIDS | CHRONIC OBSTRUCTIVE PULMONARY DISEASE, UNSPECIFIED |
| CHR T & A DISEASE NOS | MILD INTERMITTENT ASTHMA, UNCOMPLICATED |
| PERITONSILLAR ABSCESS | MILD INTERMITTENT ASTHMA WITH (ACUTE) EXACERBATION |
| CHRONIC LARYNGITIS | MILD INTERMITTENT ASTHMA WITH STATUS ASTHMATICUS |
| CHRONIC LARYNGOTRACHEITIS | MILD PERSISTENT ASTHMA, UNCOMPLICATED |
| RHINITIS DUE TO POLLEN | MILD PERSISTENT ASTHMA WITH (ACUTE) EXACERBATION |
| ALLERGIC RHINITIS DUE TO ANIMAL (CAT) (DOG) HAIR AND DANDER | MODERATE PERSISTENT ASTHMA, UNCOMPLICATED |
| ALLERGIC RHINITIS DUE TO OTHER ALLERGEN | MODERATE PERSISTENT ASTHMA WITH (ACUTE) EXACERBATION |
| ALLERGIC RHINITIS, CAUSE UNSPECIFIED | MODERATE PERSISTENT ASTHMA WITH STATUS ASTHMATICUS |
| HYPERTROPHY OF NASAL TURBINATES | SEVERE PERSISTENT ASTHMA, UNCOMPLICATED |
| OTH NASAL & SINUS DIS | SEVERE PERSISTENT ASTHMA WITH (ACUTE) EXACERBATION |
| NASAL MUCOSITIS | SEVERE PERSISTENT ASTHMA WITH STATUS ASTHMATICUS |
| OTHER DISEASE OF NASAL CAVITY AND SINUSES | UNSPECIFIED ASTHMA WITH (ACUTE) EXACERBATION |
| UNSPECIFIED DISEASE OF PHARYNX | UNSPECIFIED ASTHMA WITH STATUS ASTHMATICUS |
| PHARYNGEAL CELLULITIS | UNSPECIFIED ASTHMA, UNCOMPLICATED |
| PARAPHARYNGEAL ABSCESS | EXERCISE INDUCED BRONCHOSPASM |
| RETROPHARYNGEAL ABSCESS | COUGH VARIANT ASTHMA |
| PHAR/NASOPHARYNX EDEMA | OTHER ASTHMA |
| PHARYNX/NASOPHARYNX CYST | BRONCHIECTASIS WITH ACUTE LOWER RESPIRATORY INFECTION |
| OTHER DISEASES OF PHARYNX, NOT ELSEWHERE CLASSIFIED | BRONCHIECTASIS WITH (ACUTE) EXACERBATION |
| VOCAL CORD PARALYSIS NOS | BRONCHIECTASIS, UNCOMPLICATED |
| UNILAT PART PARAL V/C | ACUTE RESPIRATORY DISTRESS SYNDROME |
| UNILAT COMPL PARAL V/C | ACUTE PULMONARY EDEMA |
| BILAT PART PARAL V/C | CHRONIC PULMONARY EDEMA |
| BILAT COMPL PARAL V/C | PULMONARY EOSINOPHILIA, NOT ELSEWHERE CLASSIFIED |
| POLYP OF VOCAL CORD OR LARYNX | ALVEOLAR PROTEINOSIS |
| VOCAL CORD DISEASE NEC | IDIOPATHIC PULMONARY HEMOSIDEROSIS |
| EDEMA OF LARYNX | PULMONARY FIBROSIS, UNSPECIFIED |
| UNSPECIFIED DISEASE OF LARYNX | IDIOPATHIC INTERSTITIAL PNEUMONIA, NOT OTHERWISE SPECIFIED |
| STENOSIS OF LARYNX | IDIOPATHIC PULMONARY FIBROSIS |
| LARYNGEAL SPASM | IDIOPATHIC NON-SPECIFIC INTERSTITIAL PNEUMONITIS |
| OTHER DISEASES OF LARYNX, NOT ELSEWHERE CLASSIFIED | ACUTE INTERSTITIAL PNEUMONITIS |
| URT HYPERSENSIT RXN NOS | RESPIRATORY BRONCHIOLITIS INTERSTITIAL LUNG DISEASE |
| UPPER RESP DIS NEC & NOS | CRYPTOGENIC ORGANIZING PNEUMONIA |
| ADENOVIRAL PNEUMONIA | DESQUAMATIVE INTERSTITIAL PNEUMONIA |
| RSV PNEUMONIA | LYMPHOID INTERSTITIAL PNEUMONIA |
| PARAINFLUENZA VIR PNEUM | LYMPHANGIOLEIOMYOMATOSIS |
| VIRAL PNEUMONIA NEC | ADULT PULMONARY LANGERHANS CELL HISTIOCYTOSIS |
| VIRAL PNEUMONIA, UNSPECIFIED | SURFACTANT MUTATIONS OF THE LUNG |
| PNEUMONIA IN CMV DISEASE | OTHER INTERSTITIAL LUNG DISEASES OF CHILDHOOD |
| PNEUM IN ASPERGILLOSIS | OTHER SPECIFIED INTERSTITIAL PULMONARY DISEASES |
| PNEUMONIA IN OTHER SYSTEMIC MYCOSES | INTERSTITIAL PULMONARY DISEASE, UNSPECIFIED |
| PNEUM IN INFECT DIS NEC | GANGRENE AND NECROSIS OF LUNG |
| PNEUMONIA, ORGANISM UNSPECIFIED | ABSCESS OF LUNG WITH PNEUMONIA |
| INFLUENZA W PNEUMONIA | ABSCESS OF LUNG WITHOUT PNEUMONIA |
| INFLUENZA WITH OTHER RESPIRATORY MANIFESTATIONS | ABSCESS OF MEDIASTINUM |
| INFLUENZA WITH OTHER MANIFESTATIONS | PYOTHORAX WITH FISTULA |
| FLU D/T CERTAIN VIRUSES | PYOTHORAX WITHOUT FISTULA |
| INFLUENZA D/T AIV | PLEURAL EFFUSION, NOT ELSEWHERE CLASSIFIED |
| FLU D/T AIV W PNEUMONIA | MALIGNANT PLEURAL EFFUSION |
| FLU D/T AIV W RESP NEC | PLEURAL EFFUSION IN OTHER CONDITIONS CLASSIFIED ELSEWHERE |
| INFLUENZA D/T 2009 H1N1 | PLEURAL PLAQUE WITH PRESENCE OF ASBESTOS |
| FLU 2009 H1N1 W RESP NEC | PLEURAL PLAQUE WITHOUT ASBESTOS |
| FLU NOV INFL A W PNEUM | SPONTANEOUS TENSION PNEUMOTHORAX |
| FLU NOV INFL A-RESP NEC | OTHER PNEUMOTHORAX |
| FLU NOV INFL A-MAN NEC | PNEUMOTHORAX, UNSPECIFIED |
| BRONCHITIS, NOT SPECIFIED AS ACUTE OR CHRONIC | FIBROTHORAX |
| SIMPLE CHR BRONCHITIS | HEMOTHORAX |
| MUCOPURULENT CHRONIC BRONCHITIS | OTHER SPECIFIED PLEURAL CONDITIONS |
| OCB W/O EXACERBATION | PLEURAL CONDITION, UNSPECIFIED |
| OBSTRUCTIVE CHRONIC BRONCHITIS WITH (ACUTE) EXACERBATION | ACUTE RESPIRATORY FAILURE, UNSPECIFIED WHETHER WITH HYPOXIA OR HYPERCAPNIA |
| OBSTRUCTIVE CHRONIC BRONCHITIS WITH ACUTE BRONCHITIS | ACUTE RESPIRATORY FAILURE WITH HYPOXIA |
| CHRONIC BRONCHITIS NEC | ACUTE RESPIRATORY FAILURE WITH HYPERCAPNIA |
| UNSPECIFIED CHRONIC BRONCHITIS | CHRONIC RESPIRATORY FAILURE, UNSPECIFIED WHETHER WITH HYPOXIA OR HYPERCAPNIA |
| EMPHYSEMATOUS BLEB | CHRONIC RESPIRATORY FAILURE WITH HYPOXIA |
| OTHER EMPHYSEMA | CHRONIC RESPIRATORY FAILURE WITH HYPERCAPNIA |
| EXTRINSIC ASTHMA, UNSPECIFIED | ACUTE AND CHRONIC RESPIRATORY FAILURE, UNSPECIFIED WHETHER WITH HYPOXIA OR HYPERCAPNIA |
| EXTR ASTHMA W EXACER | ACUTE AND CHRONIC RESPIRATORY FAILURE WITH HYPOXIA |
| INTRINSIC ASTHMA, UNSPECIFIED | ACUTE AND CHRONIC RESPIRATORY FAILURE WITH HYPERCAPNIA |
| INTR ASTHMA W EXACER | RESPIRATORY FAILURE, UNSPECIFIED, UNSPECIFIED WHETHER WITH HYPOXIA OR HYPERCAPNIA |
| CHRONIC OBSTRUCTIVE ASTHMA, UNSPECIFIED | RESPIRATORY FAILURE, UNSPECIFIED WITH HYPOXIA |
| CHR OBSTR ASTH W EXACER | RESPIRATORY FAILURE, UNSPECIFIED WITH HYPERCAPNIA |
| EXERCISE INDUCED BRONCHOSPASM | ACUTE BRONCHOSPASM |
| COUGH VARIANT ASTHMA | OTHER DISEASES OF BRONCHUS, NOT ELSEWHERE CLASSIFIED |
| ASTHMA, UNSPECIFIED TYPE, UNSPECIFIED | ATELECTASIS |
| ASTHMA, UNSPECIFIED TYPE, WITH STATUS ASTHMATICUS | INTERSTITIAL EMPHYSEMA |
| ASTHMA, UNSPECIFIED TYPE, WITH (ACUTE) EXACERBATION | OTHER DISORDERS OF LUNG |
| BRONCHIECT W/O AC EXACER | DISEASES OF MEDIASTINUM, NOT ELSEWHERE CLASSIFIED |
| BRONCHIECTASIS WITH ACUTE EXACERBATION | MEDIASTINITIS |
| FARMERS' LUNG | MEDIASTINAL MASS |
| ALL ALVEOLITIS/PNEUM NOS | DISORDERS OF DIAPHRAGM |
| CHRONIC AIRWAY OBSTR NEC | OTHER SPECIFIED RESPIRATORY DISORDERS |
| EMPYEMA W FISTULA | RESPIRATORY DISORDER, UNSPECIFIED |
| EMPYEMA WITHOUT MENTION OF FISTULA | RESPIRATORY DISORDERS IN DISEASES CLASSIFIED ELSEWHERE |
| PLEURISY WITHOUT MENTION OF EFFUSION OR CURRENT TUBERCULOSIS | ACUTE SINUSITIS |
| PLEURISY WITH EFFUSION, WITH MENTION OF A BACTERIAL CAUSE OTHER THAN TUBERCULOSIS | AC LARYNGITIS/TRACHEITIS |
| MALIGNANT PLEURAL EFFUSION | ACUTE TRACHEITIS |
| OTH PLEURAL EFFUS NOT TB | ACUTE LARYNGOTRACHEITIS |
| UNSPECIFIED PLEURAL EFFUSION | AC LARYNGOTRACH W OBSTR |
| SPONT TENS PNEUMOTHORAX | ACUTE EPIGLOTTITIS |
| IATROGENIC PNEUMOTHORAX | SUPRAGLOTTITIS NOS |
| PNEUMOTHORAX NEC | AC URI MULT SITES/NOS |
| ABSCESS OF LUNG | ACUTE URI MULT SITES NEC |
| MEDIASTINUM ABSCESS | AC BRONCHITIS/BRONCHIOL |
| PULMONARY CONGESTION AND HYPOSTASIS | NASAL POLYPS |
| POSTINFLAMMATORY PULMONARY FIBROSIS | POLYPOID SINUS DEGEN |
| PULMON ALVEO PROTEINOSIS | CHR PHARYN/NASOPHARYNG |
| IPH | CHRONIC SINUSITIS |
| IDIO INTERST PNEUM NOS | CHR T & A DISEASE |
| IDIOPATHIC PULM FIBROSIS | T & A HYPERTROPHY |
| IDIO NONSP INTERST PNEUM | ADENOID VEGETATIONS |
| ACUTE INTERST PNEUM | CHR LARYNG/LARYNGOTRACH |
| RESP BRONCHIOL ILD | ALLERGIC RHINITIS |
| CRYPTOGEN ORGANIZ PNEUM | RHINITIS DUE TO FOOD |
| DESQUAM INTERST PNEUM | OTH UP RESPIRATORY DIS |
| LYMPHANGIOLEIOMYOMATOSIS | DISEASE OF PHARYNX NEC |
| ADULT PLCH | OTHER DISEASE OF LARYNX |
| LUNG SURFACT MUTATIONS | LARYNGEAL CELLULITIS |
| ILD OF CHILDHOOD NEC | VIRAL PNEUMONIA |
| OTHER SPECIFIED ALVEOLAR AND PARIETOALVEOLAR PNEUMONOPATHIES | SARS PNEUMONIA |
| SYST SCLEROSIS LUNG DIS | PNEUMONIA ORGANISM NEC |
| ACUTE CHEST SYNDROME | PNEUMONIA D/T ORG NEC |
| LUNG INVOLVEMENT IN OTHER DISEASES CLASSIFIED ELSEWHERE | PNEUM IN OTH INF DIS |
| PULMONARY COLLAPSE | INFLUENZA |
| INTERSTITIAL EMPHYSEMA | FLU D/T AIV W MANIF NEC |
| PULMONARY EOSINOPHILIA | FLU 2009 H1N1 W PNEUM |
| ACUTE LUNG EDEMA NOS | FLU 2009 H1N1 W MAN NEC |
| ALLERGIC BRONCHOPULMONARY ASPERGILLOSIS | FLU D/T NOV INFLUENZA A |
| ACUTE RESPIRATORY FAILURE | EXTRINSIC ASTHMA |
| OTHER PULMONARY INSUFF | EXTRINSIC ASTHMA WITH STATUS ASTHMATICUS |
| CHR RESPIRATORY FAILURE | INTRINSIC ASTHMA |
| AC & CHR RESP FAILURE | INTRINSIC ASTHMA WITH STATUS ASTHMATICUS |
| OTHER DISEASES OF LUNG, NOT ELSEWHERE CLASSIFIED | CHR OBSTRUCTIVE ASTHMA |
| ACUTE BRONCHOSPASM | CHR OBSTR ASTH W STATUS |
| OTHER DISEASES OF TRACHEA AND BRONCHUS | OTHER FORMS ASTHMA |
| MEDIASTINITIS | UNSPECIFIED ASTHMA |
| DISORDERS OF DIAPHRAGM | EXTR ALLERGIC ALVEOLITIS |
| OTHER DISEASES OF RESPIRATORY SYSTEM, NOT ELSEWHERE CLASSIFIED | BAGASSOSIS |
| UNSPECIFIED DISEASE OF RESPIRATORY SYSTEM | BIRD-FANCIERS' LUNG |
| ACUTE NASOPHARYNGITIS /COMMON COLD | SUBEROSIS |
| ACUTE MAXILLARY SINUSITIS, UNSPECIFIED | MALT WORKERS' LUNG |
| ACUTE RECURRENT MAXILLARY SINUSITIS | MUSHROOM WORKERS' LUNG |
| ACUTE FRONTAL SINUSITIS, UNSPECIFIED | MAPLE BARK-STRIPPER LUNG |
| ACUTE RECURRENT FRONTAL SINUSITIS | "VENTILATION" PNEUMONIT |
| ACUTE ETHMOIDAL SINUSITIS, UNSPECIFIED | ALL ALVEOLITIS/PNEUM NEC |
| ACUTE RECURRENT ETHMOIDAL SINUSITIS | EMPYEMA |
| ACUTE SPHENOIDAL SINUSITIS, UNSPECIFIED | PLEURISY |
| ACUTE RECURRENT SPHENOIDAL SINUSITIS | POSTOP AIR LEAK |
| ACUTE PANSINUSITIS, UNSPECIFIED | PRIM SPON PNEUMOTHORAX |
| ACUTE RECURRENT PANSINUSITIS | 2ND SPON PNEUMOTHORAX |
| ACUTE SINUSITIS, UNSPECIFIED | CHRONIC PNEUMOTHORAX |
| ACUTE RECURRENT SINUSITIS, UNSPECIFIED | AIR LEAK NEC |
| STREPTOCOCCAL PHARYNGITIS | LUNG/MEDIASTINUM ABSCESS |
| ACUTE PHARYNGITIS, UNSPECIFIED | OTH ALVEO PNEUMONOPATHY |
| ACUTE STREPTOCOCCAL TONSILLITIS, UNSPECIFIED | PULMON ALVEO MICROLITH |
| ACUTE RECURRENT STREPTOCOCCAL TONSILLITIS | IDIO LYMPH INTERST PNEUM |
| ACUTE TONSILLITIS, UNSPECIFIED | OTHER ILD OF CHILDHOOD |
| ACUTE RECURRENT TONSILLITIS, UNSPECIFIED | NCH OF INFANCY |
| ACUTE LARYNGITIS | PUL INTERST GLYCOGENOSIS |
| ACUTE TRACHEITIS WITHOUT OBSTRUCTION | ALV CAPILL DYSP W VN MIS |
| ACUTE TRACHEITIS WITH OBSTRUCTION | ALVEO PNEUMONOPATHY NOS |
| ACUTE LARYNGOTRACHEITIS | LUNG INVOLV IN DCE |
| SUPRAGLOTTITIS, UNSPECIFIED, WITHOUT OBSTRUCTION | RHEUMATIC PNEUMONIA |
| SUPRAGLOTTITIS, UNSPECIFIED, WITH OBSTRUCTION | OTHER LUNG DISEASES |
| ACUTE OBSTRUCTIVE LARYNGITIS / CROUP | COMPENSATORY EMPHYSEMA |
| ACUTE EPIGLOTTITIS WITHOUT OBSTRUCTION | LUNG DISEASE NEC |
| ACUTE EPIGLOTTITIS WITH OBSTRUCTION | MEDIASTINUM DISEASE NEC |
| ACUTE LARYNGOPHARYNGITIS | OTHER ACUTE SINUSITIS |
| ACUTE UPPER RESPIRATORY INFECTION, UNSPECIFIED | OTHER ACUTE RECURRENT SINUSITIS |
| INFLUENZA DUE TO IDENTIFIED NOVEL INFLUENZA A VIRUS WITH PNEUMONIA | ACUTE PHARYNGITIS DUE TO OTHER SPECIFIED ORGANISMS |
| INFLUENZA DUE TO IDENTIFIED NOVEL INFLUENZA A VIRUS WITH OTHER RESPIRATORY MANIFESTATIONS | ACUTE TONSILLITIS DUE TO OTHER SPECIFIED ORGANISMS |
| INFLUENZA DUE TO IDENTIFIED NOVEL INFLUENZA A VIRUS WITH OTHER MANIFESTATIONS | ACUTE RECURRENT TONSILLITIS DUE TO OTHER SPECIFIED ORGANISMS |
| INFLUENZA DUE TO OTHER IDENTIFIED INFLUENZA VIRUS WITH OTHER RESPIRATORY MANIFESTATIONS | INFLUENZA DUE TO IDENTIFIED NOVEL INFLUENZA A VIRUS WITH GASTROINTESTINAL MANIFESTATIONS |
| INFLUENZA DUE TO UNIDENTIFIED INFLUENZA VIRUS WITH UNSPECIFIED TYPE OF PNEUMONIA | INFLUENZA DUE TO OTHER IDENTIFIED INFLUENZA VIRUS WITH UNSPECIFIED TYPE OF PNEUMONIA |
| INFLUENZA DUE TO UNIDENTIFIED INFLUENZA VIRUS WITH SPECIFIED PNEUMONIA | INFLUENZA DUE TO OTHER IDENTIFIED INFLUENZA VIRUS WITH THE SAME OTHER IDENTIFIED INFLUENZA VIRUS PNE |
| INFLUENZA DUE TO UNIDENTIFIED INFLUENZA VIRUS WITH OTHER RESPIRATORY MANIFESTATIONS | INFLUENZA DUE TO OTHER IDENTIFIED INFLUENZA VIRUS WITH OTHER SPECIFIED PNEUMONIA |
| INFLUENZA DUE TO UNIDENTIFIED INFLUENZA VIRUS WITH GASTROINTESTINAL MANIFESTATIONS | INFLUENZA DUE TO OTHER IDENTIFIED INFLUENZA VIRUS WITH GASTROINTESTINAL MANIFESTATIONS |
| INFLUENZA DUE TO UNIDENTIFIED INFLUENZA VIRUS WITH OTITIS MEDIA | INFLUENZA DUE TO OTHER IDENTIFIED INFLUENZA VIRUS WITH ENCEPHALOPATHY |
| ADENOVIRAL PNEUMONIA | INFLUENZA DUE TO OTHER IDENTIFIED INFLUENZA VIRUS WITH MYOCARDITIS |
| RESPIRATORY SYNCYTIAL VIRUS PNEUMONIA | INFLUENZA DUE TO OTHER IDENTIFIED INFLUENZA VIRUS WITH OTITIS MEDIA |
| PARAINFLUENZA VIRUS PNEUMONIA | INFLUENZA DUE TO OTHER IDENTIFIED INFLUENZA VIRUS WITH OTHER MANIFESTATIONS |
| HUMAN METAPNEUMOVIRUS PNEUMONIA | INFLUENZA DUE TO UNIDENTIFIED INFLUENZA VIRUS WITH ENCEPHALOPATHY |
| OTHER VIRAL PNEUMONIA | INFLUENZA DUE TO UNIDENTIFIED INFLUENZA VIRUS WITH MYOCARDITIS |
| VIRAL PNEUMONIA, UNSPECIFIED | INFLUENZA DUE TO UNIDENTIFIED INFLUENZA VIRUS WITH OTHER MANIFESTATIONS |
| BRONCHOPNEUMONIA, UNSPECIFIED ORGANISM | PNEUMONIA DUE TO SARS-ASSOCIATED CORONAVIRUS |
| LOBAR PNEUMONIA, UNSPECIFIED ORGANISM | PNEUMONIA IN DISEASES CLASSIFIED ELSEWHERE |
| PNEUMONIA, UNSPECIFIED ORGANISM | HYPOSTATIC PNEUMONIA, UNSPECIFIED ORGANISM |
| ACUTE BRONCHITIS DUE TO HEMOPHILUS INFLUENZAE | OTHER PNEUMONIA, UNSPECIFIED ORGANISM |
| ACUTE BRONCHITIS DUE TO PARAINFLUENZA VIRUS | ACUTE BRONCHITIS DUE TO COXSACKIEVIRUS |
| ACUTE BRONCHITIS DUE TO RESPIRATORY SYNCYTIAL VIRUS | ACUTE BRONCHITIS DUE TO ECHOVIRUS |
| ACUTE BRONCHITIS DUE TO RHINOVIRUS | ACUTE BRONCHIOLITIS DUE TO HUMAN METAPNEUMOVIRUS |
| ACUTE BRONCHITIS DUE TO OTHER SPECIFIED ORGANISMS | ACUTE BRONCHIOLITIS DUE TO OTHER SPECIFIED ORGANISMS |
| ACUTE BRONCHITIS, UNSPECIFIED | ALLERGIC RHINITIS DUE TO FOOD |
| ACUTE BRONCHIOLITIS DUE TO RESPIRATORY SYNCYTIAL VIRUS | OTHER CHRONIC SINUSITIS |
| ACUTE BRONCHIOLITIS, UNSPECIFIED | POLYPOID SINUS DEGENERATION |
| UNSPECIFIED ACUTE LOWER RESPIRATORY INFECTION | MIXED SIMPLE AND MUCOPURULENT CHRONIC BRONCHITIS |
| VASOMOTOR RHINITIS | UNILATERAL PULMONARY EMPHYSEMA /MACLEOD'S SYNDROME |
| ALLERGIC RHINITIS DUE TO POLLEN | PANLOBULAR EMPHYSEMA |
| OTHER SEASONAL ALLERGIC RHINITIS | OTHER EMPHYSEMA |
| ALLERGIC RHINITIS DUE TO ANIMAL (CAT) (DOG) HAIR AND DANDER | MILD PERSISTENT ASTHMA WITH STATUS ASTHMATICUS |
| OTHER ALLERGIC RHINITIS | PULMONARY ALVEOLAR MICROLITHIASIS |
| ALLERGIC RHINITIS, UNSPECIFIED | OTHER ALVEOLAR AND PARIETO-ALVEOLAR CONDITIONS |
| CHRONIC RHINITIS | OTHER INTERSTITIAL PULMONARY DISEASES WITH FIBROSIS IN DISEASES CLASSIFIED ELSEWHERE |
| CHRONIC NASOPHARYNGITIS | NEUROENDOCRINE CELL HYPERPLASIA OF INFANCY |
| CHRONIC PHARYNGITIS | PULMONARY INTERSTITIAL GLYCOGENOSIS |
| CHRONIC MAXILLARY SINUSITIS | ALVEOLAR CAPILLARY DYSPLASIA WITH VEIN MISALIGNMENT |
| CHRONIC FRONTAL SINUSITIS | PRIMARY SPONTANEOUS PNEUMOTHORAX |
| CHRONIC ETHMOIDAL SINUSITIS | SECONDARY SPONTANEOUS PNEUMOTHORAX |
| CHRONIC SPHENOIDAL SINUSITIS | CHRONIC PNEUMOTHORAX |
| CHRONIC PANSINUSITIS | OTHER AIR LEAK |
| CHRONIC SINUSITIS, UNSPECIFIED | CHYLOUS EFFUSION |
| POLYP OF NASAL CAVITY | OTHER PULMONARY COLLAPSE |
| OTHER POLYP OF SINUS | COMPENSATORY EMPHYSEMA |
| NASAL POLYP, UNSPECIFIED |  |

**Table S2**: Respiratory diagnoses without an indication of a bacterial infection.
